# Appearance of a “Whip-Like” Rash in a Young Male Undergoing Therapy for Testicular Embryonal Carcinoma

**DOI:** 10.7759/cureus.33065

**Published:** 2022-12-28

**Authors:** Christian Karime, Alison Bruce, Winston Tan

**Affiliations:** 1 Department of Internal Medicine, Mayo Clinic, Jacksonville, USA; 2 Department of Dermatology, Mayo Clinic, Jacksonville, USA; 3 Department of Hematology and Oncology, Mayo Clinic, Jacksonville, USA

**Keywords:** clinical case report, chemotherapy-related toxicity, flagellated dermatitis, testicular cancer, bleomycin side effect, bleomycin

## Abstract

Bleomycin, a cytotoxic antibiotic commonly used as part of combination chemotherapy regimes in the treatment of germ cell tumors, is well known among clinicians for its potential pulmonary toxicity. Less well known, although postulated to occur through a similar pathomechanism, are the wide spectrum of dermatologic adverse reactions associated with bleomycin therapy. The current case report describes the sudden and distressing appearance of a pruritic erythematous flagellate “whip-like” rash in a 30-year-old Caucasian male undergoing treatment for testicular embryonal carcinoma following the second infusion of bleomycin-containing chemotherapy. Diagnosed as bleomycin-induced flagellate dermatitis, the case describes the dermatologic sequela and therapeutic interventions utilized. While commonly used in testicular cancer, decreasing use of bleomycin-containing chemotherapy regimens has made the appearance of this increasingly rare, yet important and distressing, toxic adverse reaction a diagnostic challenge. Given that patients may present acutely to primary care providers, dermatologists, and nurse practitioners, awareness of this rare adverse reaction is important in order to alleviate patient anxiety, initiate appropriate therapy, set expectations of expected dermal sequela, and initiate an informed collaborative discussion regarding the continuation versus cessation of bleomycin-containing therapy.

## Introduction

Bleomycin is a cytotoxic antibiotic commonly used as a part of combination chemotherapy regimes in the treatment of lymphomas, testicular and ovarian germ cell tumors, and squamous cell carcinomas of the head and neck region [1]. The cytotoxic effect of bleomycin results from intercalation and generation of activated oxygen-free radicals, which induces single and double-stranded DNA breaks and halts DNA replication in the G2 phase [2]. Up to 70% of bleomycin is excreted unchanged in the urine, while the remainder is inactivated by the enzyme bleomycin hydrolase within tissues. As such, reduced activity of this enzyme in lung tissue and skin has been associated with the predominant pulmonary and cutaneous toxicity of bleomycin [1,3,4].

The dermatologic toxicity of bleomycin includes a wide spectrum, including Raynaud’s phenomenon, elbow and knee hyperkeratosis, nailbed changes, peeling of skin on palmar and plantar surfaces, digital gangrene, and pigmentary alterations [1,5]. Flagellate dermatitis is a less common but unique dermatologic toxicity of bleomycin, with the sudden appearance of cutaneous erythematous linear macular streaks as though the patient has been whipped [1]. In this report, we highlight a case of non-resolving flagellate dermatitis in a young male with testicular embryonal carcinoma treated with bleomycin-containing chemotherapy.

## Case presentation

**Figure 1:**
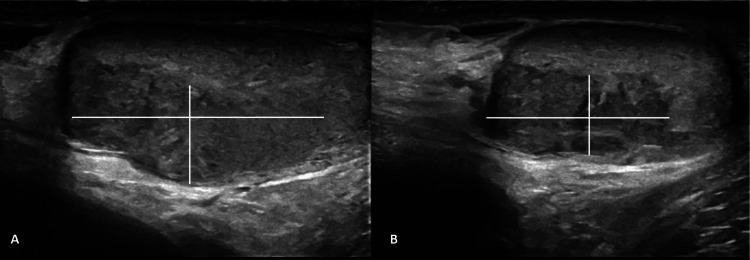
Ultrasound of right testicle illustrating a well-demarcated homogenous intratesticular mass measuring 3.4 x 2.4 x 1.4 cm A: sagittal view; B: transverse view

A 30-year-old Caucasian male presented to the emergency room with a history of intermittent right-sided testicular pain and enlargement, which was first noted approximately one year earlier. On physical examination, the patient was noted to have unilateral testicular enlargement with a palpable mass. Sonographic imaging revealed a right well-demarcated homogenous intratesticular mass measuring 3.4 x 2.4 x 1.4 cm suspicious for testicular neoplasm (Figure 1). The patient subsequently underwent right radical orchiectomy, with post-operative histopathology confirming stage 2C right testicular embryonal carcinoma with lymphovascular and spermatic cord invasion. Subsequent contrast-enhanced computer tomography (CECT) of the chest and abdomen noted retroperitoneal lymphadenopathy, with enlarged lymph nodes located posterior to the duodenum (2.2 x 2.6 x 4.4cm) and adjacent to the inferior vena cava and abdominal aorta (1.8 x 1.9 x 2.8 cm and 1.3 x 1.3 x 1.1cm, respectively). Upon consultation with Medical Oncology, the patient was planned to undergo treatment with three cycles of the chemotherapy combination of bleomycin, etoposide, and cisplatin (commonly referred to as BEP). Baseline laboratory tests revealed normal renal function (creatinine 0.95, estimated glomerular filtration rate >90) and unremarkable chest imaging.

**Figure 2:**
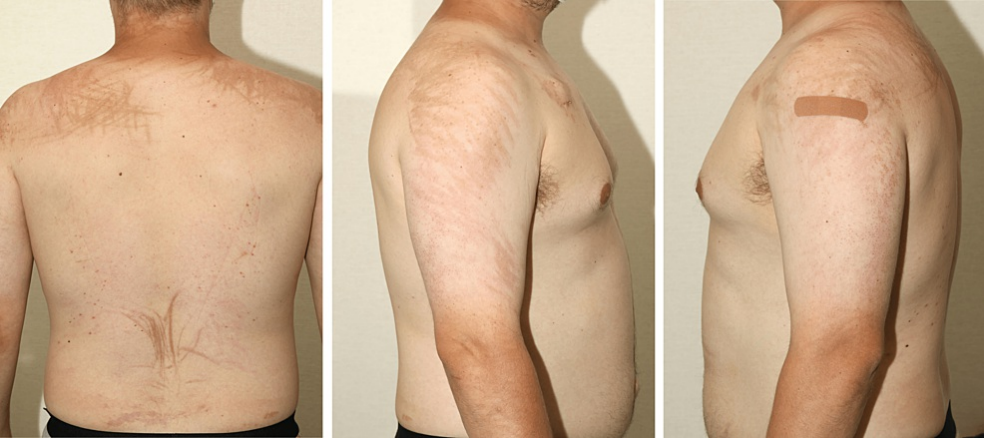
Bleomycin-induced flagellate dermatitis Hyperpigmented red-to-brown streaking rash resembling whip marks is seen on the upper and mid back as well as shoulders bilaterally, consistent with bleomycin-induced flagellate dermatitis.

On day 9, approximately one hour after receiving the second dose of bleomycin, the patient developed a pruritic and burning erythematous rash on the upper torso and shoulders. Whilst the patient expressed significant initial distress, the initial sensation of burning and pruritus subsided within hours without intervention and the patient did not seek medical attention. On follow-up with Medical Oncology seven days later, the patient was noted to have a persistent non-pruritic red-to-brown streak-like rash on the upper torso and shoulders. Due to the unfamiliar nature of dermal findings and proximity to chemotherapy administration, bleomycin infusion was paused while a further dermatologic assessment was sought. Detailed evaluation at the Department of Dermatology noted persistent non-pruritic, hyperpigmented, brown, elongated linear macules on the upper and mid back, arms, and thighs without oral involvement (Figure 2). The patient denied systemic symptoms as well as any personal or family history of dermatologic disorders or allergies. Given the temporal relationship with bleomycin infusion and characteristic flagellated whip-like appearance, the dermatologic findings were deemed consistent with bleomycin-induced flagellate dermatitis. No dermal biopsy was pursued at the time given patient preference. Treatment with daily oral cetirizine 10 mg and topical fluocinonide 0.05% cream was initiated, with oral diphenhydramine provided as needed for pruritus. Given the rare adverse reaction with no available definite treatment, a collaborative discussion was initiated between the patient, Dermatology, and Medical Oncology regarding the risk-benefit of continuation versus cessation of bleomycin chemotherapy. Full BEP chemotherapy with bleomycin was elected to resume with curative intent and close monitoring by both Dermatology and Medical Oncology, with the patient completing all intended chemotherapy cycles.

Upon subsequent evaluation three months after the initial rash appearance, the patient was noted to have persistent flagellated dermatitis with hyperpigmented dark-brown streaks on the upper back, arms, and thighs despite self-reported adherence to medication regimen of oral cetirizine and topical fluocinonide. Whilst no significant increase in total rash extent was seen, streak-like lesions were found to have deepened in pigmentation. Of note, the patient reported increased sun exposure over the preceding days to weeks. Repeat CECT three months after completion of BEP therapy demonstrated the persistent appearance of previously noted retroperitoneal lymphadenopathy, but of significantly reduced size. No new foci of concern or bleomycin-associated pulmonary toxicity were noted. Currently, at nine months after completion of BEP therapy, the patient remains in partial remission with between 60-80% decrease in size of retroperitoneal lymph nodes. The patient continues to follow closely with both Dermatology and Medical Oncology.

## Discussion

Testicular cancer is one of the most common solid organ malignancies in young males, with the mainstay of therapy involving radial orchiectomy and adjuvant therapy (surveillance, radiotherapy, or chemotherapy) depending on clinical stage. For patients with Stage 2C embryonal carcinoma (non-seminoma), the recommended treatment includes three cycles of full BEP therapy or four cycles of etoposide and cisplatin only (EP), with EP considered in patients aged over 50 years or in patients with renal impairment [6]. The reported incidence of bleomycin-induced flagellated dermatitis is estimated to be between 8-20% [5,7]. While commonly used in the treatment of testicular cancer, the overall declining usage of bleomycin-containing chemotherapy regimens has made this rare cutaneous adverse reaction increasingly infrequently observed in everyday clinical practice [5,7]. Most reports of cases of bleomycin-induced flagellate dermatitis describe a prodrome of either localized or generalized pruritus, with linear erythematous macular lesions appearing on the upper torso and limbs as though the patient had been repeatedly whipped from multiple angles [3]. As the rash becomes less erythematous and pruritus resolves, the affected areas become red-brown and deeply pigmented [3]. Although pruritus is common and noted in our current patient, cases without a pruritic prodrome have been reported [1,8]. Consistent with the findings of the current case, the physical exam of patients with flagellated dermatitis usually reveals linear intermingled pigmented streaks formed by rows of adjoining thin linear macules papules. In some instances, streaks may show evidence of punctuate hemorrhages, pustules, or formation of adjoining firm papules [5]. The time of rash appearance varies significantly, ranging from several hours to several months after initial bleomycin infusion [3,5].

While flagellate dermatitis was initially believed to be related to cumulative bleomycin toxicity within the dermis, the literature supports a dose-independent relationship with initial occurrence of bleomycin-induced flagellate dermatitis at doses as low as 5 IU and as high as 465 IU [5,9]. Moreover, the literature suggests that flagellate dermatitis is independent of both the malignant disease being treated and the route of bleomycin administration, with reactions being reported after intravenous, subcutaneous, and pleural administration [3,5]. Other chemotherapeutic agents have also been associated with flagellate dermatitis, including peplomycin, docetaxel, and bendamustine [10,11]. Peplomycin, a bleomycin derivative with reduced pulmonary toxicity, is used in lymphomas and prostatic carcinomas and has been implicated in flagellated dermatitis by similar mechanisms to bleomycin [12]. Furthermore, the taxane, docetaxel, has been reported to cause flagellate dermatitis with dermal characteristics and distribution resembling bleomycin [13]. Of note, while docetaxel is known to have a high rate of cutaneous reactions in up to 70% of patients, flagellate dermatitis is uncommon [13]. Similar to bleomycin, reports suggest that docetaxel-induced flagellate dermatitis can be suppressed by corticosteroid administration [10]. Finally, there is one report in the literature on bendamustine-induced flagellate dermatitis through yet unclear mechanisms [11]. In relation to non-chemotherapeutic agents, consumption of undercooked shiitake mushrooms has been found to occasionally cause the appearance of a flagellate rash. This is thought to be caused by polysaccharide instability at high temperatures. However, shiitake mushroom consumption has not been associated with longstanding post-inflammatory hyperpigmentation [14]. Other conditions that are associated with findings of a flagellate rash include dermatomyositis, adult-onset Still's disease, and human immunodeficiency virus infection [3,9].

Histological and ultra-structural studies indicate that bleomycin reduces epidermal turnover, resulting in prolonged contact between melanocytes and keratinocytes [8,15]. Additional histological features, albeit broad and non-specific, are thought to depend on the stage of rash evolution when biopsied. An extensive summary of histological features can be found in the work of Ziemer et al., with salient features including lymphocytic and eosinophilic dermal infiltrate, vacuolar degeneration of basal melanocytes, acantholysis and basal layer hyperpigmentation [9]. While the etiology of bleomycin-induced flagellate dermatitis and hyperpigmentation remains unclear, several probable mechanisms have been proposed, including; increased melanogenesis, pigmentary incontinence secondary to inflammation, and toxic cutaneous effects of bleomycin itself [15]. It is postulated that increased local release of histamine and vasodilation secondary to a dermographic stimulus (such as scratching) may lead to excessive dermal accumulation of bleomycin without effective means of hydrolase-mediated degradation [7,9]. This accumulation has been suggested to further increase the release of proinflammatory cytokines and non-immunogenic degranulation of mast cells [9]. However, given that a pruritic prodrome has not been universally reported in cases of bleomycin-induced flagellate dermatitis, the role of a dermographic-inflammatory process remains unclear [8]. Additionally, as bleomycin is predominantly excreted unchanged in the urine, patients with renal impairment may have a significantly reduced bleomycin clearance and thus perhaps are predisposed to potential dermatologic toxicity [4]. Previous research has shown that renal impairment is one of the most important factors for the development of pulmonary toxicity; however, to date, no such association has been reported in the case of flagellate dermatitis including our current case [16].

While there is no specific treatment available for bleomycin-induced flagellated dermatitis, evidence suggests that the use of oral antihistamines in combination with topical and/or oral corticosteroids is of benefit. Nevertheless, long-lasting post-inflammatory hyperpigmentation in the affected area is a common sequel [5,9]. While discontinuation of bleomycin can be considered in cases of severe rash or in regimes where omission does not interfere with overall therapeutic success, a clinical risk-benefit analysis may be warranted in milder cases or when bleomycin is considered central to therapy. Previous reports suggest that while pigmentary changes may persist for between 6-12 months, most cases are reversible following the discontinuation of bleomycin. However, bleomycin re-exposure may cause both rash extension and recurrence [3,17]. In two previously described cases of bleomycin-induced flagellate dermatitis after initial treatment, rash reoccurrence ensued on re-exposure to bleomycin despite the interim resolution. In both cases, stopping bleomycin resulted in rash resolution without reoccurrence [9,18]. Further reports have demonstrated that continued bleomycin therapy despite the appearance of flagellate dermatitis may significantly worsen pruritus and dermal findings [5]. Nevertheless, the literature suggests that even though long-lasting hyperpigmentation may occur, bleomycin-induced flagellate dermatitis can be controlled with antihistamines and corticosteroids and, hence, does not necessarily require bleomycin discontinuation [9]. In the current case, the continuation of bleomycin-based chemotherapy was chosen after shared decision-making with the patient and family due to the primary curative intent of treatment. In our current case, the continuation of bleomycin was not associated with rash size increase during the follow-up period. Nevertheless, the patient was noted to have darkened pigmentation at the three-month follow-up appointment. It remains unclear if this hyperpigmentation was secondary to the continuation of bleomycin chemotherapy or due to increased sunlight exposure in the weeks prior. Indeed, direct sun exposure has been known to worsen post-inflammatory hyperpigmentation, and thus it is conceivable that sunlight may have had a similar effect on hyperpigmentation associated with bleomycin-induced flagellate dermatitis [19].

## Conclusions

The present case describes the sudden appearance of a pruritic flagellate rash in a 30-year-old male undergoing chemotherapy with bleomycin for testicular embryonal carcinoma. While the sudden appearance of a non-resolving pruritic rash is understandably frightening for patients undergoing chemotherapy, flagellate dermatitis is a benign occasional cutaneous adverse effect of bleomycin. Unlike pulmonary manifestations, flagellate dermatitis has not been associated with increased morbidity or mortality and may not necessitate bleomycin discontinuation. Given that patients may present acutely to primary care providers, dermatologists, and oncologists with this unique dermatologic manifestation of bleomycin, it is important that healthcare providers are aware of this adverse cutaneous reaction in order to alleviate anxiety, initiate therapy, set appropriate patient expectations, and initiate an informed collaborative discussion regarding the continuation versus cessation of bleomycin-containing chemotherapy treatment. 
